# *Cryptosporidium Parvum* Genome Project

**DOI:** 10.1002/cfg.67

**Published:** 2001-02

**Authors:** Mitchell S. Abrahamsen

**Affiliations:** Veterinary PathoBiology University of Minnesota 1988 Fitch Avenue St. Paul MN 55108 USA

## Abstract

A lack of basic understanding of parasite biology has been a limiting factor in designing effective means of treating and preventing disease caused by *Cryptosporidium parvum.* Since the genomic DNA sequence encodes all of the heritable information responsible for development, disease pathogenesis, virulence, species permissiveness and immune resistance, a comprehensive knowledge of the *C. parvum* genome will provide the necessary information required for cost-effective and targeted research into disease prevention and treatment. With the recent advances in high-throughput automated DNA sequencing capabilities, large-scale genomic sequencing has become a cost-effective and time-efficient approach to understanding the biology of an organism. In addition, the continued development and implementation of new software tools that can scan raw sequences for signs of genes and then identify clues as to potential functions, has provided the final realization of the potential rewards of genome sequencing. To further our understanding of *C. parvum* biology, we have initiated a random shotgun sequencing approach to obtain the complete sequence of the IOWA isolate of *C. parvum*. Our progress to date has demonstrated that sequencing of the *C. parvum* genome will be an efficient and costeffective method for gene discovery of this important eukaryotic pathogen. This will allow for the identification of key metabolic and immunological features of the organism that will provide the basis for future development of safe and effective strategies for prevention and treatment of disease in AIDS patients, as well as immunocompetent hosts. Moreover, by obtaining the complete sequence of the *C. parvum* genome, effective methods for subspecific differentiation (strain typing) and epidemiologic surveillance (strain tracking) of this pathogen can be developed.


**Comparative and Functional Genomics**
*Comp Funct Genom* 2001; **2**: 19–21.

**Conference Paper**

### *Cryptosporidium parvum* genome project

Mitchell S. Abrahamsen*
*Veterinary PathoBiology, University of Minnesota, St. Paul, MN, USA*

**Correspondence to:
M. S. Abrahamsen, Veterinary PathoBiology, University of Minnesota, 1988 Fitch Avenue, St. Paul, MN 55108, USA.
E-mail: abe@umn.edu*

### Abstract

**A lack of basic understanding of parasite biology has been a limiting factor in designing effective means of treating and preventing disease caused by *Cryptosporidium parvum*. Since the genomic DNA sequence encodes all of the heritable information responsible for development, disease pathogenesis, virulence, species permissiveness and immune resistance, a comprehensive knowledge of the *C. parvum* genome will provide the necessary information required for cost-effective and targeted research into disease prevention and treatment. With the recent advances in high-throughput automated DNA sequencing capabilities, large-scale genomic sequencing has become a cost-effective and time-efficient approach to understanding the biology of an organism. In addition, the continued development and implementation of new software tools that can scan raw sequences for signs of genes and then identify clues as to potential functions, has provided the final realization of the potential rewards of genome sequencing. To further our understanding of *C. parvum* biology, we have initiated a random shotgun sequencing approach to obtain the complete sequence of the IOWA isolate of *C. parvum*. Our progress to date has demonstrated that sequencing of the *C. parvum* genome will be an efficient and cost-effective method for gene discovery of this important eukaryotic pathogen. This will allow for the identification of key metabolic and immunological features of the organism that will provide the basis for future development of safe and effective strategies for prevention and treatment of disease in AIDS patients, as well as immunocompetent hosts. Moreover, by obtaining the complete sequence of the *C. parvum* genome, effective methods for subspecific differentiation (strain typing) and epidemiologic surveillance (strain tracking) of this pathogen can be developed.** **Copyright © 2001 John Wiley & Sons, Ltd.**

**Keywords:** ***Cryptosporidium parvum*; apicomplexa; genome; sequencing**

*Cryptosporidium parvum* is a well-recognized cause of diarrhoea in humans and animals throughout the world, and is associated with a substantial degree of morbidity and mortality in patients with the acquired immunodeficiency syndrome (AIDS). At the present time, there is no effective therapy for treating or preventing infection with *C. parvum*. This is primarily due to a lack of understanding of the basic cellular and molecular biology of this pathogen in terms of virulence factors, genome structure, gene expression and gene regulation.

*C. parvum* is an obligate intracellular protozoan that progresses sequentially through a complex life cycle involving both asexual and sexual developmental stages. A major limitation to understanding the basic biology of *C. parvum* is the inability to obtain purified samples of the various asexual, sexual and intracellular developmental stages of the parasite. As a consequence, few biochemical studies have been directed toward understanding the complicated developmental biology of *C. parvum* or the unique biochemistry and molecular mechanisms involved during host–parasite interactions. Considering the absolute dependence of *Cryptosporidium* on the host cell for development of the multiple asexual and sexual life cycle stages, it is crucial that we have a better understanding of the biology of intracellular *Cryptosporidium* development and the unique biochemistry and molecular mechanisms involved in host–parasite interactions. These are likely to represent some of the most unique aspects of *Cryptosporidium* biology that will

Copyright © 2001 John Wiley & Sons, Ltd.

provide the basis for future development of safe and effective strategies for prevention and treatment.

Recently, several large-scale sequencing efforts have been initiated to further our understanding of *C. parvum* biology. These include an expressed sequence tag (EST) project characterizing gene expression in *C. parvum* sporozoites [3], and the generation of a limited number of genome survey sequences (GSSs) [2,3]. In addition to the identification of large numbers of *C. parvum* genes unique to any currently in the sequence databases, these efforts have identified numerous expressed sequences with similarities to known genes of other organisms. These studies demonstrate that large-scale sequencing of the *C. parvum* genome is an efficient and cost-effective method for gene discovery for this important eukaryotic pathogen.

Since the genomic DNA sequence encodes all of the heritable information responsible for development, disease pathogenesis, virulence, species permissiveness and immune resistance, a comprehensive knowledge of the *C. parvum* genome will provide the necessary information required for cost-effective and targeted research into disease prevention and treatment. This type of approach for *C. parvum* gene discovery is not subject to the same limitations as an EST approach. All genes can be identified, irrespective of their developmental expression. In addition, by having the entire genomic sequence, the complete open reading frame for each gene can be identified based on primary structure, not homology, as is the case for a GSS approach.

An important issue to consider when proposing to obtain the genomic sequence of any organism, and particularly eukaryotic organisms, is the organization and size of the genome. Improved separation of *C. parvum* chromosomes using contour-clamped homogeneous electric fields combined with scanning densitometry has determined that the most likely number of chromosomes is eight [1]. The chromosomes range in size from 1.03 Mb to 1.54 Mb with a total genome size of $\sim$10.4 Mb. This is a relatively small eukaryotic genome that makes it an ideal candidate for a genome-sequencing approach. In addition, the vast majority of *C. parvum* genes characterized to date lack introns. As is the case for bacterial genome projects, this makes data analysis straightforward and increases the efficiency of characterizing the coding sequences of putative *C. parvum* genes.

To further our understanding of this important pathogen, we have undertaken the task of sequencing the entire genome of the IOWA isolate of *C. parvum*. This is being accomplished using a random sequence analysis of the *C. parvum* genome at seven-fold coverage of genomic fragments ($>$99% theoretical completion) in double-stranded plasmid vectors. A considerable saving of effort can be achieved if the inserts are greater than twice the average sequence read length, so that each template can be sequenced from both ends without redundancy. Sequencing from both ends greatly facilitates orientating contigs and determining the final genome structure. Three plasmid libraries were constructed using DNA fragments randomly sheared by nebulization in the 2.0–2.5 kb, 2.5–3.0 kb and 3.0–5.0 kb size range. The use of different size ranges of inserts ensures the most complete representation of the *C. parvum* genome and will provide the necessary templates to assist in the gap-closure process.

Currently, 28 000 templates have been sequenced, generating $>$2.5 Mb of DNA sequence, or approximately 2.5-fold coverage of the genome. These sequences have been assembled into 3286 contigs using Phrap of the PHRED/PHRAP/CONSED analysis software that was developed at the University of Washington (**http://bozeman.mbt.washington.edu/**). Of these contigs, $\sim$1200 have a length $>$2000 bp, with total contig length of $>$7 Mb. These contigs represent $\sim$70% of the *C. parvum* genome, with the number of contigs beginning to decrease as the assembly process continues (progress and project information is available at **http://www.cbc.umn.edu/ResearchProjects/AGAC/Cp/index.htm**). Importantly, analysis of the available *C. parvum* sequences has revealed a low frequency of repetitive sequences (estimated to be $<$1% of the genome) and an estimated overall (A+T) content of $\sim$65%. Thus it appears that the *C. parvum* genome will not be subject to many of the technical difficulties associated with sequencing of other protozoan pathogens.

Completion of the *C. parvum* genome will provide a vast amount of information regarding the biology of *C. parvum* that will have many long-term benefits. In addition to identifying all of the *C. parvum* genes independent of their developmental expression or transcript level, the studies outlined in this proposal will provide other valuable information and genetic tools for studying the

Copyright © 2001 John Wiley & Sons, Ltd.

biology of this important pathogenic protozoan. Currently, there exists a need for better genetic markers to be used in molecular epidemiological studies of *C. parvum* transmission and population dynamics. Useful highly polymorphic genetic markers that have been identified in other eukaryotic organisms are almost exclusively found in non-transcribed regions of the genome. By having the complete sequence of the *C. parvum* genome, all putative genes will be identified, allowing the non-transcribed regions to be identified and characterized to determine whether they contain useful genetic markers. Furthermore, the data generated by these studies will provide sequence information on the promoters of *C. parvum* genes, the structural organization of the genome, and the basis for future comparative genome analysis with other eukaryotic organisms. Each one of these issues represents a substantial advance in our understanding of *C. parvum* biology and will provide the foundation for future research efforts.

Taking full advantage of the information contained within the *C. parvum* genome will require a careful, systematic analysis of the sequences and the application of a wide variety of computer methods. Comparisons with complete genomic and chromosome sequences from other apicomplexan protozoans, as well as additional small eukaryotes, will undoubtedly shed light on many of the complex biochemical processes important for obligate intracellular pathogens. Ultimately, a better understanding of the complicated biochemical pathways of *C. parvum* will be gained by exploiting the combined genomic, biological and genetic information available from all of the experimental coccidian models. In particular, comparative analysis of the ongoing sequencing projects and experimental genetic models available in *Toxoplasma* and *Plasmodium* will be invaluable for future studies to address specific issues of coccidian development and host–parasite communication.

### References

1. Blunt DS, Khramtsov NV, Upton SJ, Montelone BA. 1997. Molecular karyotype analysis of *Cryptosporidium parvum*: evidence for eight chromosomes and a low-molecular-size molecule. *Clin Diag Lab Immunol* **4**: 11–13.
2. Liu C, Vigdorovich V, Kapur V, Abrahamsen MS. 1999. A random survey of the *Cryptospordium parvum* genome. *Infect Immun* **67**: 3960–3969.
3. Strong WB, Nelson RG. 2000. Preliminary profile of the *Cryptosporidium parvum* genome: an expressed sequence tag and genome survey sequence analysis. *Mol Biochem Parasitol* **107**: 1–32.

Copyright © 2001 John Wiley & Sons, Ltd.
